# Resting-state EEG power and coherence vary between migraine phases

**DOI:** 10.1186/s10194-016-0697-7

**Published:** 2016-11-02

**Authors:** Zehong Cao, Chin-Teng Lin, Chun-Hsiang Chuang, Kuan-Lin Lai, Albert C. Yang, Jong-Ling Fuh, Shuu-Jiun Wang

**Affiliations:** 1 Faculty of Engineering and Information Technology, University of Technology Sydney, Sydney, Australia; 2 Department of Electrical and Computer Engineering, Institute of Electrical Control Engineering, National Chiao Tung University, Hsinchu, Taiwan; 3 Brain Research Center, National Chiao Tung University, Hsinchu, Taiwan; 4 Neurological Institute, Taipei Veterans General Hospital, Taipei, Taiwan; 5 Department of Psychiatry, Taipei Veterans General Hospital, Taipei, Taiwan; 6 Faculty of Medicine, National Yang-Ming University School of Medicine, Taipei, Taiwan; 7 Division of Interdisciplinary Medicine and Biotechnology, Beth Israel Deaconess Medical Center/Harvard Medical School, Boston, MA USA; 8 Brain Research Center, National Yang-Ming University, Taipei, Taiwan

**Keywords:** Migraine without aura, Resting-state, EEG, Power, Isolated effective coherence

## Abstract

**Background:**

Migraine is characterized by a series of phases (inter-ictal, pre-ictal, ictal, and post-ictal). It is of great interest whether resting-state electroencephalography (EEG) is differentiable between these phases.

**Methods:**

We compared resting-state EEG energy intensity and effective connectivity in different migraine phases using EEG power and coherence analyses in patients with migraine without aura as compared with healthy controls (HCs). EEG power and isolated effective coherence of delta (1–3.5 Hz), theta (4–7.5 Hz), alpha (8–12.5 Hz), and beta (13–30 Hz) bands were calculated in the frontal, central, temporal, parietal, and occipital regions.

**Results:**

Fifty patients with episodic migraine (1–5 headache days/month) and 20 HCs completed the study. Patients were classified into inter-ictal, pre-ictal, ictal, and post-ictal phases (*n* = 22, 12, 8, 8, respectively), using 36-h criteria. Compared to HCs, inter-ictal and ictal patients, but not pre- or post-ictal patients, had lower EEG power and coherence, except for a higher effective connectivity in fronto-occipital network in inter-ictal patients (*p* < .05). Compared to data obtained from the inter-ictal group, EEG power and coherence were increased in the pre-ictal group, with the exception of a lower effective connectivity in fronto-occipital network (*p* < .05). Inter-ictal and ictal patients had decreased EEG power and coherence relative to HCs, which were “normalized” in the pre-ictal or post-ictal groups.

**Conclusion:**

Resting-state EEG power density and effective connectivity differ between migraine phases and provide an insight into the complex neurophysiology of migraine.

**Electronic supplementary material:**

The online version of this article (doi:10.1186/s10194-016-0697-7) contains supplementary material, which is available to authorized users.

## Background

Migraine is a common and potentially disabling neurological disorder that affects about 11 % of people worldwide [1], including 9.1 % in Taiwan [2]. A minority of migraine patients (13–31 %) experience aura symptoms prior to headache onset [3, 4]. Although some patients with migraine without aura exhibit other prodromal symptoms [3], their migraine attacks are generally unpredictable [5]. Taking abortive medications during the early stages of a migraine attack increases medication efficacy and reduces recurrence [6]. Therefore, pre-emptive detection of migraine attacks may be clinically beneficial, especially for patients with migraine without aura.

Although the underlying pathophysiology of migraine is still unclear, prior neurophysiological studies have shown abnormal cortical evoked potentials [7, 8] in different stimulus models of migraine, such as lacking habituation of visual and auditory cortex excitability [9] and reduced motor and visual cortical thresholds [10]. Specifically, compared to controls, migraine patients show increased phase synchronization after stimulation during the migraine-free inter-ictal phase (between post- and pre-ictal phases) [11, 12]. Furthermore, in the pre-ictal phase (before migraine attacks), migraine patients exhibit normal habituation of visually-evoked and auditory-evoked potentials [13], but decreased motor cortex activity [14]. However, resting-state cortical activities, such as EEG power density and effective connectivity, have not been studied much in relation to particular migraine phases [15, 16].

There has been growing interest in resting-state functional and effective connectivity in recent years [17]. Compared to stimulation-related tasks, resting-state experiments, in which additional cortical activations are not induced, are more convenient and comfortable for migraine patients. Previous resting-state studies have shown dynamic EEG power changes in migraine patients [15, 16, 18]. EEG coherence, which involves cross-correlation between signals in the frequency domain to reveal interrelationships of EEG signals, is a widely used measure of functional connectivity [19]. High-level coherence between two EEG signals reflects synchronized neuronal oscillations, whereas low-level coherence suggests desynchronized neural activity. Although EEG coherence analysis has been applied to study migraine-related neural abnormalities in stimulation tasks [11, 12], resting-state EEG coherence in different phases of migraine has not yet been examined. The notion of EEG-detected connectivity is supported by resting-state functional MRI studies showing significant network changes in migraine [20, 21]. Additionally, classical coherence analysis has disadvantages such as volume conduction and influences from common sources or indirect connections [17]. These are typical problems when bivariate approaches are used instead of multivariate approaches. To solve these inherent problems, new connectivity measurement, such as isolated effective coherence (iCoh) [22], has been proposed to render more accurate interactions among the cerebral regions. This study aimed to investigate dynamic changes in resting-state EEG power intensity and brain connectivity networks across different phases of migraine (inter-ictal, pre-ictal, ictal, and post-ictal). In particular, we focused on a subgroup of migraine patients with low frequency because prediction of migraine attacks in this group could have substantial clinical utilities.

## Methods

### Subjects

Patients with migraine without aura, who were diagnosed by board-certified neurologists at the Headache Clinic, Taipei Veterans General Hospital (VGH) as having low-frequency migraine (1–5 days per month) were invited to join this study. Diagnoses of migraine without aura were based on the ICHD-2 criteria [23]. Age-matched HCs were enrolled from hospital colleagues, their relatives, or friends who did not have past or family histories of migraine, nor any headache attack during the past year.

**Fig. 1:**
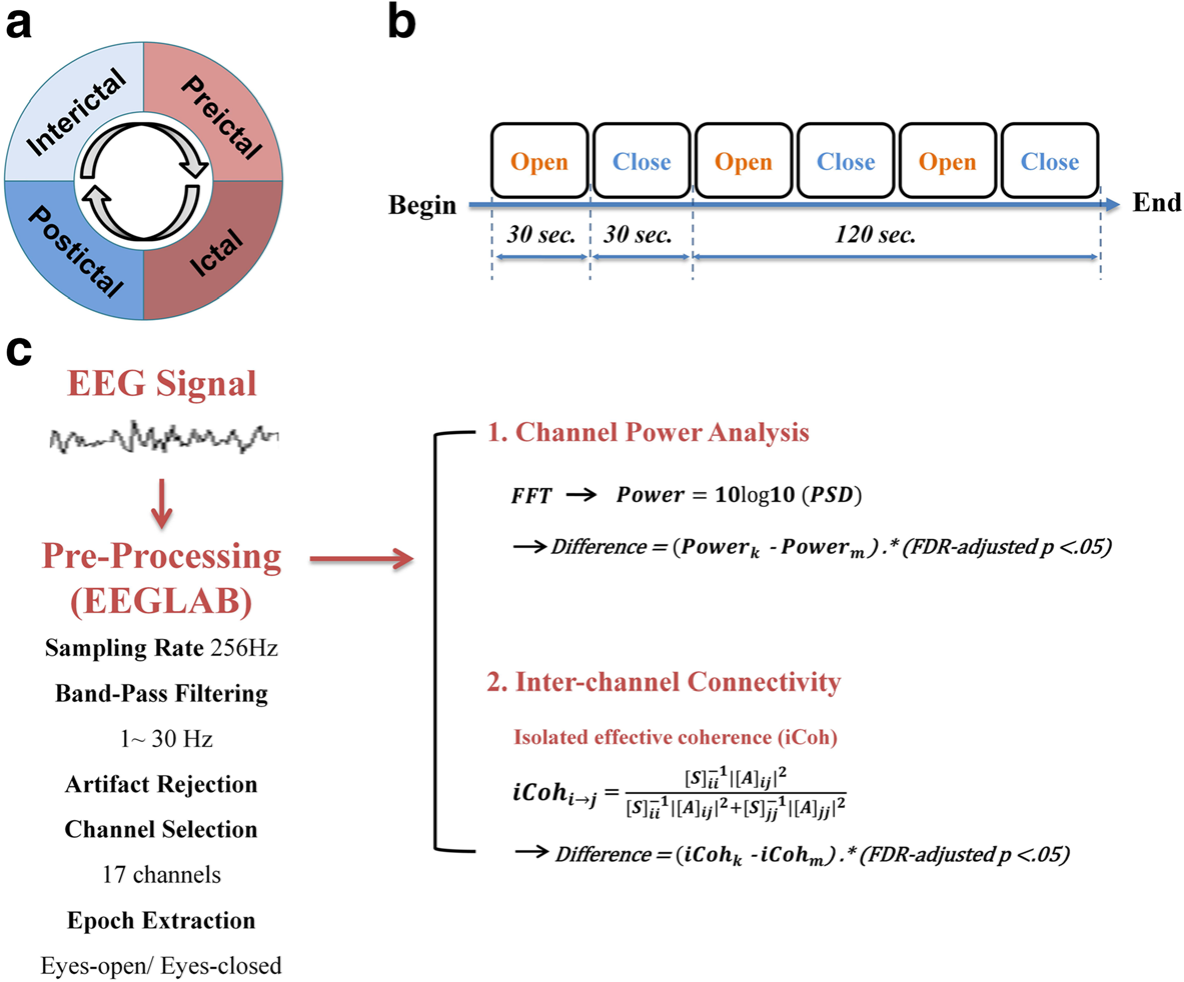
Analytical procedures. **a**: Migraine cycle; **b**: Resting-state EEG recording; **c**: EEG signal processing

Each patient kept a headache diary and completed a structured questionnaire on demographics, headache profile, medical history, and medication use. The headache profiles included the duration of migraine history (years), the severity of migraine, headache frequency (days per month), and Migraine Disability Assessment (MIDAS). In addition, the Beck Depression Inventory (BDI) and Hospital Anxiety Depression Scale (HADS) were administered to screen for psychological disturbances. On EEG study days, patients’ migraine phases were designated as inter-ictal, pre-ictal, ictal, or post-ictal based on the patients’ headache diaries (Fig. 1a). Ictal phase was coded when patients had suffered a migraine attack on the day of EEG study. Pre-ictal and post-ictal phases were coded when patients were within 36 h before or after an ictal phase on the day of EEG study, respectively. Inter-ictal phase was coded for patients in a pain-free period between pre-ictal and post-ictal phases.

Subjects were excluded if they had systemic diseases, connective tissue disorders, neurological or psychiatric disorders, as well as other painful conditions according to their self-report. All subjects had normal vision after correction. To prevent the mis-classification of migraine phases or the distracted effect on EEG, patients were requested not to take analgesics within 2 days before EEG recording, nor take any psychotropic drugs within 4 weeks before the EEG study. None of our patients were on any migraine preventive agents.

This study was approved by the Institutional Review Board at Taipei VGH (approved ID: 2011-06-009IC). Informed consent was obtained from all subjects before they joined the study.

### Experimental design

Experiments were performed in a quiet, dimly light room in our hospital. During the first 2 min of the experiment, subjects were instructed to take several deep breaths while they adapted to the environment. Next, subjects were instructed to open their eyes for 30 s and close their eyes for 30 s and to repeat this sequence for a total of three times (Fig. 1b). Meanwhile, EEG signals were recorded using Nicolet EEG system (Natus Medical, Incorporated, San Carlos, CA, USA) with Ag/AgCl electrodes. Eighteen EEG electrodes (Fp1, Fp2, F7, F3, Fz, F4, F8, T3, C3, Cz, C4, T4, T5, P3, P4, T6, O1, and O2) were placed according to the conventional 10–20 EEG system [24] and the guideline of American Clinical Neurophysiology Society [25]. Fz was used as the reference channel. The skin under the reference electrodes were abraded and disinfected with a 70 % isopropyl alcohol swab before calibration. The impedance of the electrodes was calibrated under 5 kΩ. The EEG signals were amplified and digitized at a sampling rate of 256 Hz with 16-bit quantization.

### EEG data analysis

The EEG data were analyzed with EEGLAB, an open-source MATLAB toolbox for electrophysiological signal processing and analysis [26]. The analytical procedures for EEG signal processing included a band-pass filter, artifact rejection, epoch extraction, time-frequency analysis, and coherence estimation (Fig. 1c). During signal preprocessing, raw EEG signals were subjected to 1-Hz high-pass and 30-Hz low-pass finite impulse response (FIR) filters. For the artifact rejection, firstly, apparent eye contaminations in EEG signals were manually removed by visual inspection. Secondly, Independent Component Analysis (ICA) was applied to the EEG signals and the components responsible for the eye movements and blinks were rejected. Then, the EEG signals without these artifact components was reconstructed using the back-projection method [26]. Finally, the reconstructed EEG signals were inspected again using the “Automatic Channel Rejection (ACR)” function with Kurtosis measurement and Z-score threshold of 5 to remove noisy channels. Eyes-open and eyes-closed resting-state signals of three blocks were extracted and concatenated for further analyses.

### EEG power analysis

Processed time-series data were transformed into the frequency domain by a 256-point fast Fourier transform with Welch’s method. Specifically, 90-s spans of data were analyzed with a 256-point moving window with a 128-point overlap. Windowed data were extended to 512 points by zero-padding to calculate power spectra, yielding an estimation of the power spectra with 60 frequency bins from 1 to 30 Hz (frequency resolution: 0.5 Hz). Power spectra of these windows were averaged and converted to a logarithmic scale. Mean delta (*δ*: 1–3.5 Hz), theta (*θ*: 4–7.5 Hz), alpha (*α*: 8–12.5 Hz), and beta (*β*: 13–30 Hz) band powers of 17 channels were visualized on a two-dimensional (2-D) topographic map.

### EEG coherence analysis

For all groups (inter-ictal, pre-ictal, ictal, post-ictal, and HCs), we explored the coupling between brain areas within particular frequency bands based on the up-to-date coherence algorithm, named isolated effective coherence (iCoh) [22], which is a multivariate approach to address the effective connectivity. Its advantages not only are insensitive to volume conduction but also can detect direct pathways linking brain regions. Firstly, the Source Information Flow Toolbox (SIFT) [27] in the EEGLAB was used to identify the optimal multivariate autoregressive model. Then, the magnitude of iCoh for channel $j\to$ channel $i$ at the frequency of $w$ is estimated from the following formula [22].

$$ iCo{h}_{j\to i}(w)=\frac{{\left[{S}_{\varepsilon}\right]}_{ii}^{-1}{\left|{\left[\widehat{A}(w)\right]}_{ij}\right|}^2}{{\left[{S}_{\varepsilon}\right]}_{ii}^{-1}{\left|{\left[\widehat{A}(w)\right]}_{ij}\right|}^2+{\left[{S}_{\varepsilon}\right]}_{jj}^{-1}{\left|{\left[\widehat{A}(w)\right]}_{jj}\right|}^2}, $$

where $0\le {\boldsymbol{iCoh}}_{j\to i}\;(w)\le 1$, the autoregressive coefficients ${\left[\mathbf{A}\left(\mathrm{w}\right)\right]}_{kl}\equiv 0$, for all $\left(k,\;l\right)$ such that $\left(k,\;l\right)\ne \left(i,j\right)$ and $k\ne l$ and the spectral density matrix ${\left[{\boldsymbol{S}}_{\boldsymbol{\varepsilon}}\right]}_{kl}\equiv 0$, for all $\left(k,\;l\right)$ such that $k\ne l$.

### Statistical analysis

Group differences in clinical profiles were analyzed by Student’s *t*-test (migraine patients vs. HCs) or one-way ANOVA (four phases of migraine patients) for continuous variables and chi-square or Fisher’s exact tests for categorical variables. Resting-state EEG band power and coherence values were compared across all five groups (HC and 4 migraine phase groups) by the Wilcoxon rank-sum test, followed by calculation of the false discovery rate (FDR) for multiple comparisons. The significance level was set to 0.05. Statistical analysis was performed in the SPSS software package (version 15.0) and MATLAB (2011a) Bioinformatics Toolbox.

## Results

### Demographic and clinical characteristics

A total of 61 patients with migraine without aura joined the study, of whom, 11 were excluded because of taking analgesic medications within 2 days before the EEG study, yielding a final sample of 50 patients for analysis. These 50 patients were classified into inter-ictal (*n* = 22), pre-ictal (*n* = 12), ictal (*n* = 8), and post-ictal (*n* = 8) phases. In addition, 20 HCs were also recruited. Demographic and clinical characteristics were similar between the migraine group and HC group and also similar across the four migraine phase groups (Table 1).

**Table 1:** Comparisons of demographics, headache profiles, and psychological characteristics between study groups

Characteristic	Migraine patients (*N* = 50)	HCs (*N* = 20)	*P*	Migraine phase groups				*P*
				Inter-ictal (*N* = 22)	Pre-ictal (*N* = 12)	Ictal (*N* = 8)	Post-ictal (*N* = 8)	
Sex, F:M	35:15	11:9	0.24	16:6	6:6	7:1	6:2	0.49
Age, y	36.0 ± 9.9	36.9 ± 6.7	0.63	33.0 ± 9.0	39.0 ± 7.5	40.0 ± 11.5	38.0 ± 12.4	0.27
Migraine headache profile								
Disease duration, y	16.0 ± 9.3	N/A	N/A	15.0 ± 8.1	16.0 ± 7.8	20.0 ± 9.6	16.0 ± 13.8	0.72
Frequency, d/month.	3.8 ± 1.3	N/A	N/A	3.8 ± 1.4	3.8 ± 1.3	3.6 ± 1.3	3.9 ± 1.0	0.81
Pain severity^a^	7.0 ± 1.9	N/A	N/A	8.0 ± 2.1	7.0 ± 1.8	8.0 ± 1.9	6.0 ± 1.7	0.42
MIDAS score^b^	16.3 ± 13.4	N/A	N/A	19.1 ± 16.6	17.8 ± 10.7	11.0 ± 11.8	15.7 ± 13.5	0.59
Psychometric scores								
BDI	8.7 ± 5.7	N/A	N/A	9.4 ± 6.1	7.7 ± 5.6	9.9 ± 5.3	7.9 ± 5.8	0.68
HADS-A	6.7 ± 3.7	N/A	N/A	7.6 ± 3.2	5.5 ± 3.4	6.6 ± 3.0	7.8 ± 5.7	0.41
HADS-D	4.6 ± 3.3	N/A	N/A	4.8 ± 3.4	3.6 ± 2.2	5.0 ± 2.8	4.2 ± 3.3	0.46

*Abbreviations*: *BDI* Beck Depression Inventory, *F:M* ratio of females to males, *HADS-A* Hospital Anxiety Depression Scale, Anxiety, *HADS-D* Hospital Anxiety Depression Scale, Depression, *HC* healthy controls, *MIDAS* Migraine Disability Assessment Scale. Of note, group differences in clinical profiles were analyzed by Student’s *t*-test (migraine patients vs. HCs) or one-way ANOVA (four phases of migraine patients) for continuous variables and chi-square or Fisher’s exact tests for categorical variables

^a^0–10 scale. ^b^0–270 range

### Comparisons of resting-state EEG power between migraine patients and HCs

Dynamic changes in EEG power/coherence between migraine patients and HCs or between migraine phases were more robust in the eyes-open (Figs. 2, 3, 4 and 5) than in the eyes-closed condition (Additional file 1: Figures S1, S2, S3 and S4). Therefore, we used EEG data from the eyes-open condition in subsequent analyses.

**Fig. 2:**
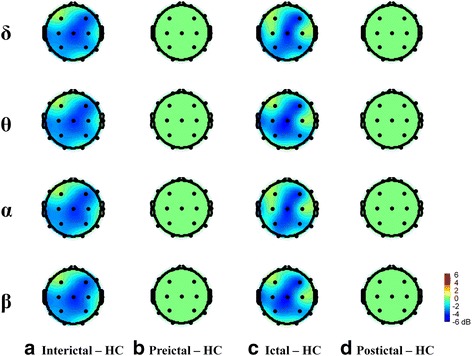
Topographical comparison of significant EEG power differences (*p* < .05) between migraine patients in different migraine phases and HCs during eyes-open recording. Color intensity indicates the magnitude of the power difference (*red* for increased power, *blue* for decreased power) in each channel

**Fig. 3:**
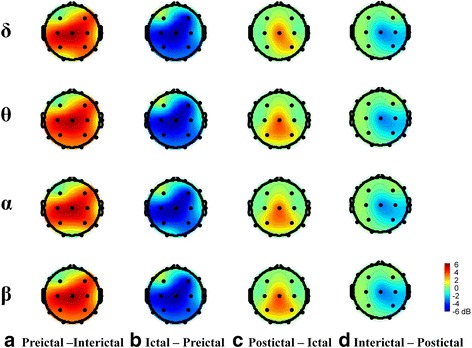
Topographical comparisons of significant EEG power differences (*p* < .05) between patients in each of the four migraine phases during eyes-open recording. Color intensity indicates the magnitude of the power difference (red for increased power, blue for decreased power) in each channel

**Fig. 4:**
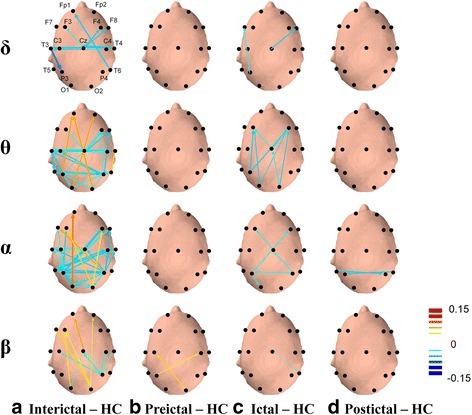
Topographical comparisons of significant EEG coherence differences (*p* < .05) between patients in different migraine phases and HCs during eyes-open recording. Line sizes and colors reflect the magnitude of the difference in coherence intensity between electrode pairs, with *red* indicating positive differences (more coherent) and *blue* indicating negative differences (more independent). The directions of *arrows* represent the direct paths of inter-channel coupling

**Fig. 5:**
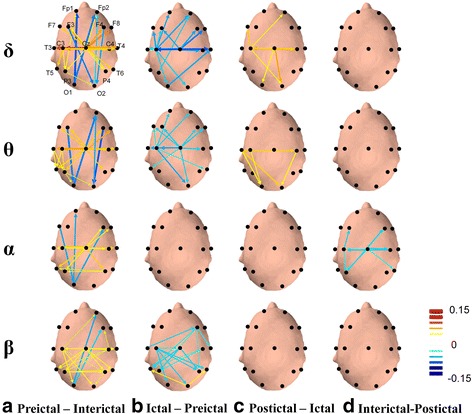
Topographical comparisons of significant EEG coherence differences (*p* < .05) between migraine patients in each of the four phases of the migraine cycle during eyes-open recording. Line sizes and colors reflect the magnitude of the difference in coherence intensity between electrode pairs, with *red* indicating positive differences (more coherent) and *blue* indicating negative differences (more independent). The directions of *arrows* represent the direct paths of inter-channel coupling

Significant differences in resting-state EEG power in the eyes-open condition in migraine patients from each phase versus HCs for the delta, theta alpha, and beta domains are shown in Fig. 2. Inter-ictal patients had significantly lower delta, theta, alpha and beta EEG power in the fronto-central (F4, C3, Cz, C4) and parietal (P3, P4) regions, compared to HCs (FDR-adjusted *p* < .05, Fig. 2a). EEG power values did not differ between pre-ictal patients and HCs in any of the four EEG frequency domains (Fig. 2b). Ictal patients had lower delta, theta, alpha and beta (fronto-central and parietal regions) power than HCs (FDR-adjusted *p* < .05, Fig. 2c). EEG power variability in post-ictal patients was similar to that in HCs (Fig. 2d).

### Comparisons of resting-state EEG power across migraine phases

Significant differences in resting-state EEG power between the four migraine-phase groups are shown in Fig. 3. EEG power intensity of pre-ictal patients in the fronto-central and parietal regions of delta theta, alpha and beta bands were higher than the corresponding values in inter-ictal patients (FDR-adjusted *p* < .05, Fig. 3a). Compared to pre-ictal patients, ictal patients had lower fronto-central and parieto-occipital delta, theta, alpha, and beta EEG power (FDR-adjusted *p* < .05, Fig. 3b). Centro-parietal delta, theta, alpha, and beta EEG power intensity were higher in post-ictal patients than in ictal patients (FDR-adjusted *p* < .05, Fig. 3c). Right centro-parietal delta, theta, alpha, and beta EEG power intensity were lower in inter-ictal patients than in post-ictal patients (FDR-adjusted *p* < .05, Fig. 3d).

### Comparisons of resting-state EEG coherence between migraine patients and HCs

Comparisons of resting-state EEG coherence between migraine patients in each phase of the migraine cycle versus HCs are shown in Fig. 4. Delta, theta, alpha, and beta EEG coherence networks were lower in inter-ictal patients than in HCs (FDR-adjusted *p* < .05; Fig. 4a), with the exception of fronto-occipital network. Specifically, inter-ictal patients had decreased delta EEG coherence in fronto-central network, theta and alpha EEG coherence in fronto-central and posterior networks, and centro-parietal reductions in beta EEG coherence. Of note, the fronto-occipital network showed enhanced EEG coherence in theta, alpha, and beta bands (FDR-adjusted *p* < .05; Fig. 4a). The EEG coherence networks of pre-ictal patients, generally, did not differ from those of HCs, except for a slight increase in posterior beta EEG coherence (Fig. 4b). The cortical connection intensities of EEG coherence networks for theta and alpha frequency bands in ictal patients were lower than those in HCs (FDR-adjusted *p* < .05; Fig. 4c). Coherence in post-ictal patients was similar to that in HCs, with the exception of a small downtrend in posterior alpha EEG coherence. (Fig. 4d).

### Comparisons of resting-state EEG coherence across migraine phases

As demonstrated in Fig. 5, significant differences in resting-state EEG coherence were observed between all pairs of consecutive migraine phases. Large significant differences in EEG coherence networks were observed in the delta, theta, and alpha bands in the frontal, central, temporal, parietal, and occipital regions. Specifically, compared to inter-ictal patients, pre-ictal patients had higher EEG coherence in the delta, theta, alpha, and beta bands (FDR-adjusted *p* < .05; Fig. 5a) except for a reduction of EEG coherence in fronto-occipital network in delta, theta, alpha and beta bands (FDR-adjusted *p* < .05; Fig. 5a). Meanwhile, ictal patients had significantly lower EEG coherence networks in the delta, theta, and beta bands than did pre-ictal patients (FDR-adjusted *p* < .05; Fig. 5b). Moreover, as in Fig. 5c, compared to ictal patients, post-ictal patients had greater EEG coherence, particularly in the delta and theta centro-occipital network (FDR-adjusted *p* < .05). Finally, compared to post-ictal patients, inter-ictal patients had markedly lower EEG coherence networks in the alpha band (FDR-adjusted *p* < .05; Fig. 5d).

## Discussion

In the present study, using resting-state EEG, we showed that migraine patients in the inter-ictal and ictal phases, but not in the pre- and post-ictal phases, exhibited lower EEG power and coherence than HCs. Comparing the phase groups in series pairs (inter-ictal, pre-ictal, ictal, post-ictal), we observed increases in EEG power and coherence from the inter-ictal to the pre-ictal phase, decreases from the pre-ictal to the ictal phase, and finally increases from the ictal to the post-ictal phase. The fronto-occipital network in inter-ictal patients showed enhanced EEG coherence as compared to HC or pre-ictal patients. Of note, our results showed higher effect sizes in the eyes-open EEG than eyes-closed EEG. The exact mechanisms are not clear. We do not know whether there is a link to the facts that visual cortical hyperexcitibility is more common in patients with migraine [28] and visual areas in eyes-open condition show greater activation than in eyes-closed condition [29].

Migraine attacks have been hypothesized to start at the cortical level [8, 30, 31]. Previously, the synchronization levels of cortical activity during visual stimulation in migraine patients have been shown to differ from those in HCs [11, 12]. Our findings provide new evidence of cortical abnormalities during a resting state as detected by EEG power spectra and coherence analyses. Furthermore, our findings complement prior resting-state EEG studies demonstrating cortical activity differences between adjacent migraine phases [15, 16].

Extending prior work showing abnormal cortical activity in migraine patients, particularly in the inter-ictal phase [8], we found that the EEG power and coherence, except for the effective connectivity in fronto-occipital network, were lower in the inter-ictal patients than in HCs. That is, migraine patients in the inter-ictal phase exhibited hypo-coupling in the frontal and centro-posterior networks, and hyper-coupling in the fronto-occipital network. Unlike our study results, previous studies showed similar EEG power between inter-ictal patients and HCs [16, 32]. Nevertheless, during the tasks for evoked potentials, inter-ictal patients have been reported to exhibit reduced EEG power [33] and synchronization [11] in relation to HCs. Compared to HCs, migraine patients showed significantly reduced EEG power and coherence during migraine attacks, which normalized after migraine attacks. These power results are in line with the results of two prior studies [34, 35]. Moreover, the decreased EEG coherence in our ictal patients suggests that hypo-coupling may occur during migraine attacks.

Our resting-state EEG results also provide information about the cortical state in the pre-ictal phase. We found significantly higher EEG power and coherence in pre-ictal versus inter-ictal phases. This increased EEG power suggests a relatively excessive cortical power intensity in the pre-ictal phase, which is generally consistent with a higher anterior delta EEG power relative to the inter-ictal phase [15]. Our elevated EEG resting-state coherence in pre-ictal phase points to hyper-coupling of regional brain connectivity, especially in the fronto- and centro-posterior networks. Intriguingly, prior studies have described a pre-ictal “normalization” (towards HCs) of cortical responses to visual and auditory evoked potentials [13, 36, 37]. The exact underlying mechanisms accounting for our findings are not known. In fact, Sakai et al. [38] demonstrated an increase or normalization of cerebral serotonin synthesis from the inter-ictal stage to migraine attacks. Nevertheless, one recent study [39] reported activation of the hypothalamus and brainstem during the prodromal phase (i.e. pre-ictal state) of migraine patients. Because our study did not employ source localization methods, the brain regions responsible for our observations in EEG power and coherence require further investigations.

This study’s major strengths were a sizable number of patients in different migraine phases and headache diary recordings for classifications of migraine phases in each patient. However, this study also had limitations. First, it is known that EEG power, concordance and coherence differences were reported in patients with psychiatric disorders, such as unipolar or bipolar disorders, as well as attention deficit hyperactivity disorder [40, 41]. We could not completely exclude the possibility that some of our participants might have such disorders since not all participants had psychiatric consultations. Second, because we recruited low-frequency episodic migraine patients only, one should be cautious about generalizing the findings to other migraine patient groups, such as high-frequency or chronic migraine patients. Third, because we employed a cross-sectional design, it is unknown whether the present results could be repeated in an examination of the same individuals with a longitudinal study design. Fourth, the number of participants and the sex ratio in each group was not fully matched. The imbalance can be explained by the low frequency of migraine attacks in our participants. The sex imbalance in different migraine cycles might be due to the small number in some cycles. Moreover, the vulnerability of coherence measures to volume conduction represents a potential confounder in our study. However, such an influence would be reduced in our study because we calculated differences only between pairs of migraine phases. Last, our study employ EEG, which records signals that are principally of cortical origin. Thus further investigations combining functional MRI with EEG should be pursued to examine the involvement of cortical/subcortical dysfunction in different migraine phases.

## Conclusions

The present study revealed dynamic changes in resting-state EEG power and effective connectivity using band power analysis and iCoh, respectively, across different migraine phases in patients with low frequency migraine. EEG effective connectivity in pre-ictal patients showed an augmented coupling in the fronto-central and centro-posterior networks and a reduced coupling in the fronto-occipital network. Such brain network dynamics could have implications for understanding complex neurophysiology of migraine before a headache attack.

## Additional file

Additional file 1: Figure S1. Topographical comparison of significant EEG power differences (*p* < .05) between migraine patients in different migraine phases and HCs during eyes-closed recording. Color intensity indicates the magnitude of the power difference (red for increased power, blue for decreased power) in each channel. **Figure S2.** Topographical comparisons of significant EEG power differences (*p* < .05) between patients in each of the four migraine phases during eyes-closed recording. Color intensity indicates the magnitude of the power difference (red for increased power, blue for decreased power) in each channel. **Figure S3.** Topographical comparisons of significant EEG coherence differences (*p* < .05) between patients in different migraine phases and HCs during eyes-closed recording. Line sizes and colors reflect the magnitude of the difference in coherence intensity between electrode pairs, with red indicating positive differences (more coherent) and blue indicating negative differences (more independent). The directions of arrow represent the direct paths of inter-channel coupling. **Figure S4.** Topographical comparisons of significant EEG coherence differences (*p* < .05) between migraine patients in each of the four phases of the migraine cycle during eyes-closed recording. Line sizes and colors reflect the magnitude of the difference in coherence intensity between electrode pairs, with red indicating positive differences (more coherent) and blue indicating negative differences (more independent). The directions of arrow represent the direct paths of inter-channel coupling. (DOCX 2036 kb)
