# Supplementary material for: Resting-state EEG power and coherence vary between migraine phases
Source: J Headache Pain. 2016 Nov 2;17(1):102. doi: 10.1186/s10194-016-0697-7 (PMC5093108; doi:10.1186/s10194-016-0697-7)

**Supplementary Materials:**

**Supplementary figure legends:**

Supplementary Figure 1. Topographical comparison of significant EEG power differences (p < .05) between migraine patients in different migraine phases and HCs during eyes-closed recording. Color intensity indicates the magnitude of the power difference (red for increased power, blue for decreased power) in each channel.

Supplementary Figure 2. Topographical comparisons of significant EEG power differences (p < .05) between patients in each of the four migraine phases during eyes-closed recording. Color intensity indicates the magnitude of the power difference (red for increased power, blue for decreased power) in each channel.

Supplementary Figure 3. Topographical comparisons of significant EEG coherence differences (p < .05) between patients in different migraine phases and HCs during eyes-closed recording. Line sizes and colors reflect the magnitude of the difference in coherence intensity between electrode pairs, with red indicating positive differences (more coherent) and blue indicating negative differences (more independent). The directions of arrow represent the direct paths of inter-channel coupling.

Supplementary Figure 4. Topographical comparisons of significant EEG coherence differences (p < .05) between migraine patients in each of the four phases of the migraine cycle during eyes-closed recording. Line sizes and colors reflect the magnitude of the difference in coherence intensity between electrode pairs, with red indicating positive differences (more coherent) and blue indicating negative differences (more independent). The directions of arrow represent the direct paths of inter-channel coupling.

**Figures:**

Supplementary Figure 1


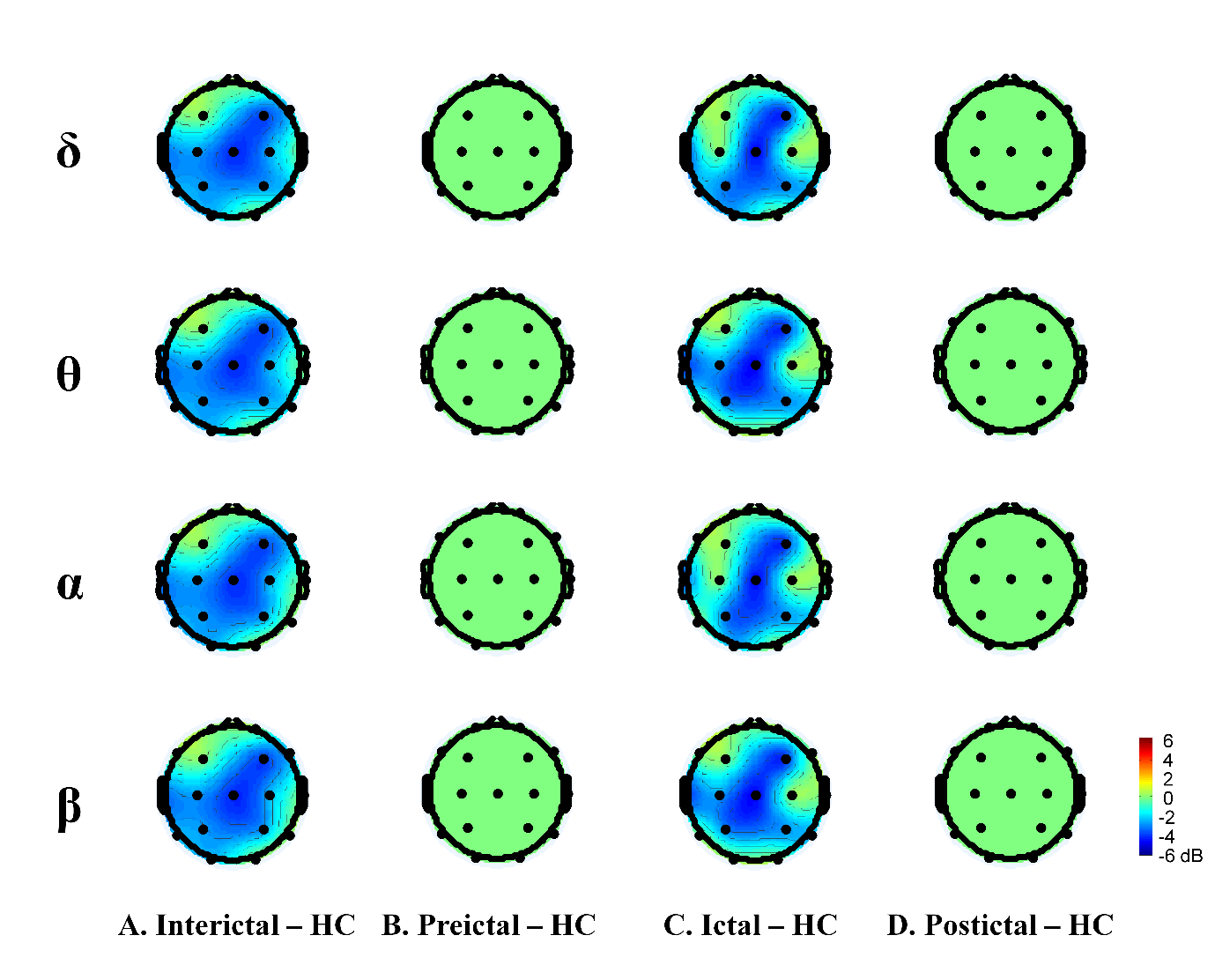


Supplementary Figure 2


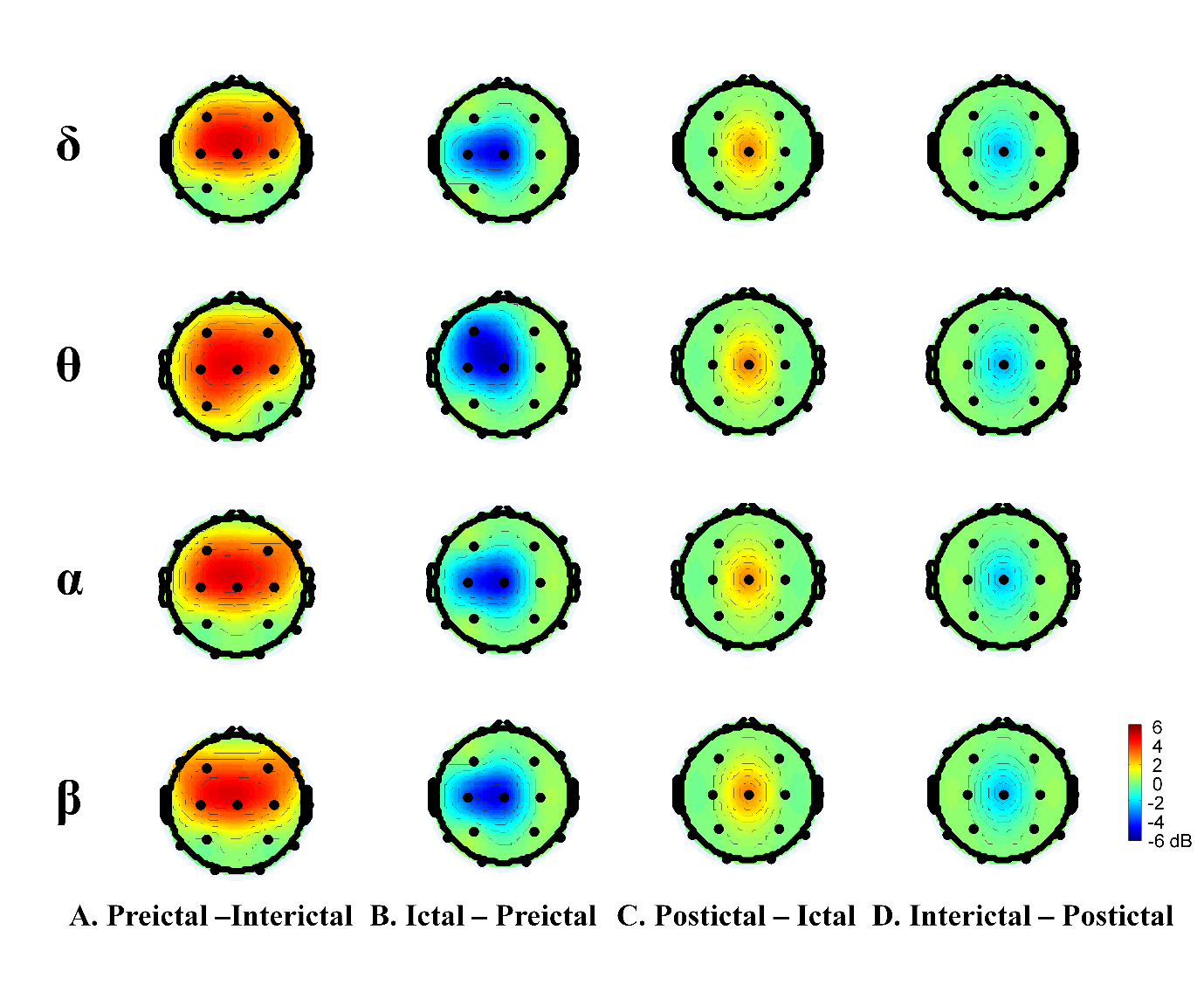


Supplementary Figure 3


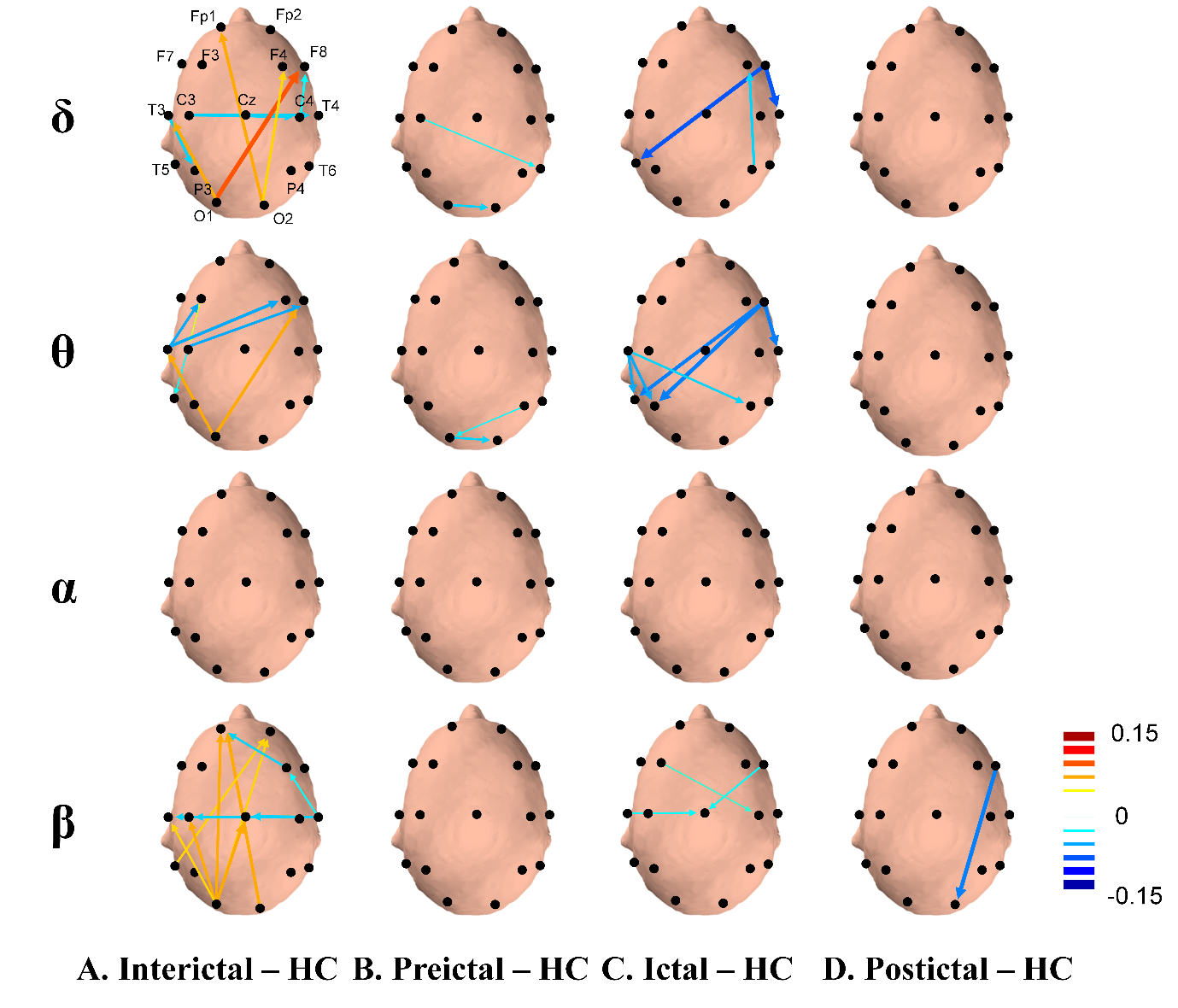


Supplementary Figure 4


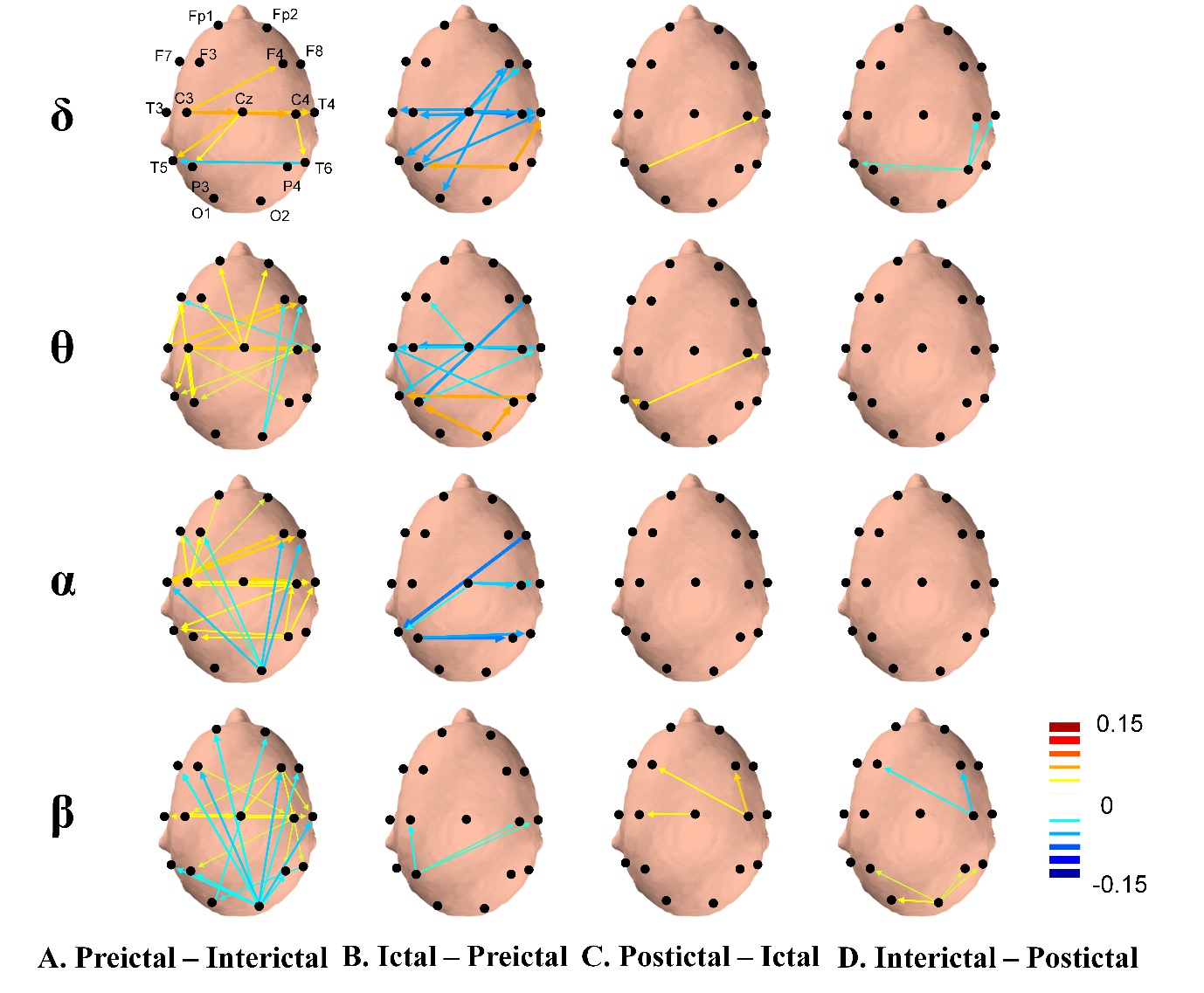

Supplement: Additional file 1: Figure S1. — Topographical comparison of significant EEG power differences (p < .05) between migraine patients in different migraine phases and HCs during eyes-closed recording. Color intensity indicates the magnitude of the power difference (red for increased power, blue for decreased power) in each channel. Figure S2. Topographical comparisons of significant EEG power differences (p < .05) between patients in each of the four migraine phases during eyes-closed recording. Color intensity indicates the magnitude of the power difference (red for increased power, blue for decreased power) in each channel. Figure S3. Topographical comparisons of significant EEG coherence differences (p < .05) between patients in different migraine phases and HCs during eyes-closed recording. Line sizes and colors reflect the magnitude of the difference in coherence intensity between electrode pairs, with red indicating positive differences (more coherent) and blue indicating negative differences (more independent). The directions of arrow represent the direct paths of inter-channel coupling. Figure S4. Topographical comparisons of significant EEG coherence differences (p < .05) between migraine patients in each of the four phases of the migraine cycle during eyes-closed recording. Line sizes and colors reflect the magnitude of the difference in coherence intensity between electrode pairs, with red indicating positive differences (more coherent) and blue indicating negative differences (more independent). The directions of arrow represent the direct paths of inter-channel coupling. (DOCX 2036 kb) [file 10194_2016_697_MOESM1_ESM.docx]
